# Financial protection analysis in eight countries in the WHO South-East Asia Region

**DOI:** 10.2471/BLT.18.209858

**Published:** 2018-07-17

**Authors:** Hui Wang, Lluis Vinyals Torres, Phyllida Travis

**Affiliations:** a World Health Organization Regional Office for South-East Asia, World Health House, Indraprastha Estate, Mahatma Gandhi Marg, New Delhi 110 002, India.

## Abstract

**Objective:**

To document the financial protection status of eight countries of the South-East Asian region and to investigate the main components of out-of-pocket expenditure on health care.

**Methods:**

We calculated two financial protection indicators using data from living standards surveys or household income and expenditure surveys in Bangladesh, Bhutan, India, Maldives, Nepal, Sri Lanka, Thailand and Timor-Leste. First, we calculated the incidence of catastrophic health expenditure, defined as the proportion of the population spending more than 10% or 25% of their total household expenditure on health. Second, using World Bank poverty lines, we determined the impoverishing effect of health-care spending by households. We also conducted an analysis of the main components of out-of-pocket expenditure.

**Results:**

Across countries in this study, 242.7 million people experienced catastrophic health expenditure at the 10% threshold, and 56.4 million at the 25% threshold. We calculated that 58.2 million people were pushed below the extreme poverty line of 1.90 United States dollars (US$) and 64.2 million people below US$ 3.10 (per capita per day values in 2011 purchasing power parity), due to out-of-pocket spending on health. Spending on medicines was the main component of out-of-pocket spending in most of the countries.

**Conclusion:**

A substantial number of people in South-East Asia experienced financial hardship due to out-of-pocket spending on health. Several countries have introduced policies to make medicines more available, but the finding that out-of-pocket expenditure on medicines remains high indicates that further action is needed to support progress towards universal health coverage.

## Résumé

**Objectif:**

Rendre compte de la situation en matière de protection financière dans huit pays de la région Asie du Sud-Est et déterminer les principales composantes des dépenses directes de soins.

**Méthodes:**

Nous avons calculé deux indicateurs de protection financière à partir de données provenant d'enquêtes sur le niveau de vie ou d'enquêtes sur les revenus et les dépenses des ménages au Bangladesh, au Bhoutan, en Inde, aux Maldives, au Népal, au Sri Lanka, en Thaïlande et au Timor-Leste. Pour commencer, nous avons calculé l'incidence des dépenses de santé catastrophiques, à savoir la part de la population qui consacrait plus de 10% ou de 25% des dépenses totales du foyer à la santé. Ensuite, à l'aide des seuils de pauvreté de la Banque mondiale, nous avons déterminé l'appauvrissement qu'entraînaient les dépenses de soins pour les ménages. Nous avons également effectué une analyse des principales composantes des dépenses directes.

**Résultats:**

Dans les pays examinés dans cette étude, 242,7 millions de personnes avaient des dépenses de santé catastrophiques au-delà du seuil de 10% et 56,4 millions au-delà de 25%. Nous avons calculé que les dépenses directes de soins poussaient 58,2 millions de personnes en-dessous du seuil de pauvreté extrême de 1,90 dollar des États-Unis ($ US) et 64,2 millions de personnes en-dessous de celui de 3,10 $ US (valeurs par habitant et par jour en parité de pouvoir d'achat de 2011). Dans la plupart des pays, l'achat de médicaments représentait la principale composante des dépenses directes.

**Conclusion:**

En Asie du Sud-Est, un nombre conséquent d'habitants a connu des difficultés financières à cause de ses dépenses directes de soins. Plusieurs pays ont mis en place des politiques visant à rendre les médicaments plus abordables, mais le fait que les dépenses directes liées à l'achat de médicaments restent élevées indique que d'autres mesures doivent être prises pour progresser en direction de la couverture sanitaire universelle.

## Resumen

**Objetivo:**

Documentar la situación de la protección financiera de ocho países de la región del Sudeste Asiático e investigar los principales componentes de los gastos directos en atención sanitaria.

**Métodos:**

Se calcularon dos indicadores de protección financiera a partir de datos de encuestas sobre el nivel de vida o los ingresos y los gastos domésticos en Bangladesh, Bhután, la India, las Maldivas, Nepal, Sri Lanka, Tailandia y Timor-Leste. En primer lugar, se calculó la incidencia del gasto catastrófico en salud, definido como la proporción de la población que gasta más del 10% o 25% del gasto doméstico total en salud. En segundo lugar, a partir de las líneas de pobreza del Banco Mundial, se determinó el efecto empobrecedor del gasto doméstico en salud. También se llevó a cabo un análisis de los principales componentes de los gastos directos.

**Resultados:**

En todos los países del estudio, 242,7 millones de personas experimentaban un gasto sanitario catastrófico en el umbral del 10%, y 56,4 millones en el umbral del 25%. Se estimó que 58,2 millones de personas se encontraban por debajo de la línea de pobreza extrema de 1,90 dólares estadounidenses (USD) y 64,2 millones de personas por debajo de los 3,10 USD (valores per cápita diarios en paridad del poder adquisitivo de 2011), debido al gasto directo en salud. El gasto en medicamentos fue el principal componente del gasto directo en la mayoría de los países.

**Conclusión:**

Un número considerable de personas del Sudeste Asiático experimentaban dificultades financieras debido al gasto directo en salud. Varios países han introducido políticas para facilitar el acceso a los medicamentos, pero la conclusión de que los gastos directos en medicamentos sigan siendo elevados indica que es necesario adoptar nuevas medidas para apoyar el progreso hacia la cobertura sanitaria universal.

## ملخص

الغرض ****توثيق حالة الحماية المالية لثمانية بلدان في منطقة جنوب شرق آسيا والتحقيق في المكونات الرئيسية لنفقات الرعاية الصحية التي تتحملها الأسر.

الطريقة قمنا بحساب مؤشرين للحماية المالية باستخدام بيانات من مسوحات مستويات المعيشة، أو مسوحات الدخل والإنفاق الأسري، في كل من بنغلاديش وبوتان والهند وملديف ونيبال وسري لانكا وتايلند وتيمور- لشتي. أولاً، قمنا بحساب معدل حدوث النفقات الصحية الجائرة، والتي تم تعريفها على أنها نسبة السكان الذين ينفقون ما يزيد عن 10٪ أو 25٪ من إجمالي نفقاتهم على الصحة. ثانياً، قمنا باستخدام الخطوط الإرشادية للفقر في البنك الدولي، وحددنا آثار الفقر المترتبة على الإنفاق للرعاية الصحية بواسطة الأسر. كما أجرينا أيضا تحليلا للمكونات الرئيسية للنفقات التي تتحملها الأسر.

النتائج ****في البلدان التي شملتها هذه الدراسة، واجه 242.7 مليون شخص نفقات صحية جائرة بنسبة 10٪، كما واجه 56.4 مليون شخص نفقات جائرة بنسبة 25٪. لقد حسبنا أن 58.2 مليون شخص قد تم دفعهم إلى ما تحت خط الفقر المدقع، والذي يبلغ 1.90 دولاراً أمريكياً (بالدولار الأمريكي)، كما تم دفع 64.2 مليون شخص إلى أقل من 3.10 دولاراً أمريكياً (وفقاً لقيم نصيب الفرد في اليوم الواحد في تعادل القوى الشرائية لعام 2011)، بسبب الإنفاق على الصحة. كان الإنفاق على الأدوية هو المكون الرئيسي للإنفاق في معظم البلدان.

الاستنتاج ****شهد عدد كبير من الأشخاص في جنوب شرق آسيا ضائقة مالية بسبب الإنفاق الشخصي على الصحة. وقد اقترحت عدة بلدان سياسات لجعل الأدوية أكثر توافراً، ولكن اكتشاف أن الإنفاق الشخصي على الأدوية لا يزال مرتفعاً يشير إلى الحاجة إلى المزيد من الإجراءات لدعم التقدم نحو التغطية الصحية الشاملة. ****

## 摘要

**目的:**

旨在记录东南亚地区八国金融保护状况,并调查医疗保健自付支出的主要组成部分。

**方法:**

我们使用不丹、东帝汶、马尔代夫、孟加拉国、尼泊尔、斯里兰卡、泰国和印度的生活标准调查或家庭收入和支出调查数据对两项金融保护指标进行计算。首先,我们计算了灾难性卫生支出的发生率,并将其定义为人口支出占其家庭卫生总支出 10% 或 25% 以上。其次,使用世界银行制定的贫困线,我们确定了家庭医疗保健支出的贫困影响。我们还对自付支出的主要组成部分进行了分析。

**结果:**

在此研究中,各国有 2.427 亿人经历了阈值 10% 的灾难性医疗支出,5640 万人经历了阈值 25% 的灾难性医疗支出。根据自付卫生支出,我们计算出 5820 万人被迫处于低于 1.90 美元 (US$) 的极端贫困线以下,6420 万人处于低于 3.10 美元的线下(2011 年购买力平价人均每日数值)。大多数国家中,药品支出是自付支出的主要组成部分。

**结论:**

由于不菲的自付卫生支出,东南亚有相当多的人处于经济困难之中。一些国家已推行更多可用药品政策,但有发现表明药品自付费用仍然很高,这说明需要采取进一步行动来推进全民医疗保险的普及。

## Резюме

**Цель:**

Документально зафиксировать статус финансовой защиты в восьми странах региона Юго-Восточной Азии и исследовать основные составляющие расходов из собственных средств на здравоохранение.

**Методы:**

Авторы рассчитали два показателя финансовой защиты, используя данные опросов для определения уровня жизни или уровня дохода семейств, а также опросов, выявляющих уровень расходов в Бангладеш, Бутане, Индии, на Мальдивских Островах, в Непале, Таиланде, Тимор-Лешти и Шри-Ланке. Сначала был рассчитан показатель частоты катастрофических расходов на здравоохранение, который определялся как доля населения, для которого расходы на здравоохранение превышали 10 или 25% от общих расходов семьи. Затем, используя установленную Всемирным банком черту бедности, авторы определили, насколько такие расходы на здравоохранение приводили к обнищанию семейств. Был также проведен анализ основных составляющих расходов из собственных средств.

**Результаты:**

Согласно данным исследования, 242,7 миллиона людей понесли катастрофические расходы на уровне 10%-го порога и 56,4 миллиона — на уровне 25%-го. Авторы подсчитали, что 58,2 миллиона людей оказались за чертой крайней нищеты, составляющей 1,90 доллара США на душу населения в день, а 64,2 миллиона оказались за чертой 3,10 доллара США на душу в день (в суммах, отражающих паритет покупательной способности на 2011 г.) по причине расходов на здравоохранение. Наиболее существенным компонентом наличных затрат во всех странах были расходы на лекарства.

**Вывод:**

Значительное количество населения в странах Юго-Восточной Азии испытывает финансовые трудности по причине наличных расходов на здравоохранение. В некоторых странах внедрены стратегии повышения доступности медикаментов, но вывод о том, что наличные расходы на лекарства по-прежнему остаются высокими, указывает на необходимость дальнейших действий в этом направлении для обеспечения всеобщего охвата услугами здравоохранения.

## Introduction

The aim of universal health coverage (UHC), as set out in *Transforming our world: the 2030 agenda for sustainable development,*^1^ is to ensure that all people and communities receive the health care they need, without experiencing financial hardship. The World Health Organization (WHO) South-East Asia Region consists of 11 Member States and almost 2 billion people living in low- and lower-middle income countries. Population health has progressively improved in recent decades, although the Region still lags behind many others, except Africa Region and fragile states elsewhere, and inequities remain.^2^ Government spending on health ranges from 0.4% to 2.5% of gross domestic product in all countries of the Region except Maldives and Thailand, lower than what has been suggested as necessary for better performance.^3^ As a result, the health financing model relies heavily on out-of-pocket expenditure by households, comprising an estimated 47% of current health expenditure on average in the Region, with a huge variation across countries from 10% to 74%.^4^ Such a high level of out-of-pocket expenditure implies a heavy financial burden on households.^5^^,^^6^ Moreover, the poor may be disproportionately affected due to fewer resources at their disposal; international evidence suggests that the costs of treatment could be prohibitively high for them to access needed health care.^7^

There are two widely used approaches to conceptualize financial hardship: (i) catastrophic spending and (ii) impoverishment. Catastrophic spending on health care occurs when out-of-pocket expenditure exceeds certain pre-defined thresholds, affecting households’ ability to spend on other necessities of life. Impoverishment refers to situations in which household spending on health pushes people into poverty. The two concepts capture different aspects of the economic consequence of out-of-pocket expenditure on households. For instance, for those whose per capita spending is just above the poverty line (threshold), a small amount of out-of-pocket expenditure on health care, although not catastrophic by definition, could lead to impoverishment. By contrast, well-off households may have catastrophic out-of-pocket expenditure, but still stay above the poverty line. Analysing both indicators is therefore important to present a fuller picture.

Efforts to develop the concept of catastrophic expenditure on health care date back to 1986. High out-of-pocket expenditure for illness, defined as a fixed amount of family income, was considered an opportunity cost both for households sacrificing consumption of other items and for societies through loss of labour productivity.^8^ Similarly, arbitrarily and exogenously defined fixed thresholds were used to define catastrophic expenditure, but instead of income, total household budget was used as the denominator.^9^ A second approach is to use capacity to pay as the denominator, which deducts the spending on necessities defined in a variety of ways (e.g. actual food expenditure,^9^ subsistence level food expenditure,^10^ maximum saturated level of expenditure on necessities,^11^ spending on food, rent and utilities^12^ and a multiple of international poverty thresholds^13^). The evolution in methods highlights the need to better differentiate the budget capacity of poor and rich households to measure the real financial impact of out-of-pocket expenditure. Previous research also underscores the difficulty in coming up with a perfect indicator that can be applicable to a wide variety of countries and surveys.

We aimed to document the financial protection status of eight countries of the WHO South-East Asian Region with the latest available data. Two indicators were calculated, the incidence of catastrophic health expenditure (indicator 3.8.2 of the sustainable development goals) and the impoverishing effect of households’ health-care spending, as defined in the joint World Health Organization and World Bank Global Monitoring reports.^14^^,^^15^ We also aimed to investigate the main components of out-of-pocket spending both at national level and by quintiles of total household expenditure.

## Methods

### Indicators

Out-of-pocket expenditure on health care is defined as payments made at the point of service, after deduction of any reimbursement. When out-of-pocket expenditure exceeds a threshold of total household budget, the household is defined as having catastrophic health spending. Suppose *m_i_* is health expenditure per household *i*, *n_i_* is total expenditure, *t_j_* is the threshold, with *t*_1_ = 10%, *t*_2_ = 25%, the catastrophic expenditure under threshold *j CHE_ij_* is 1 if *m_i_/n_i_* > *t_j_* and 0 otherwise. If we define population weight 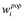 as household weight adjusted by household size, 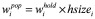, then the average incidence is defined by Equation 1 as:


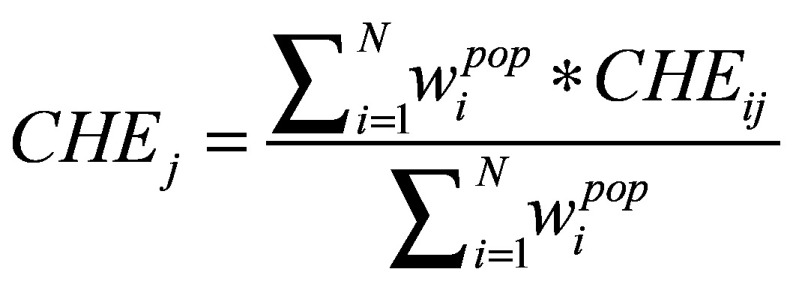
*j = 1,2*(1)

We used the change in poverty headcount ratio to calculate how many people were impoverished due to out-of-pocket expenditure. The change in poverty gap captures both the number of the households impoverished and the severity of the impoverishment. Equation 2 below defines the gross and net poverty headcount ratio and Equation 3 defines gross and net poverty gap, following previous methods.^16^ Suppose *x*_i_ is total expenditure per capita in household *i*, *PL* is the pre-defined poverty line and *c_i_* is health expenditure per capita. Then household *i* will be defined as gross-poor, or 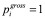, if *x*_i_ <  *PL*, or 0 otherwise; and it will be defined as net-poor, or, 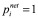, if *x*_i_−*c*_i_ <  *PL*, or 0 otherwise. Then *H^gross^*, or gross poverty headcount ratio, and *H^net^*, net headcount ratio, are defined in Equation 2:


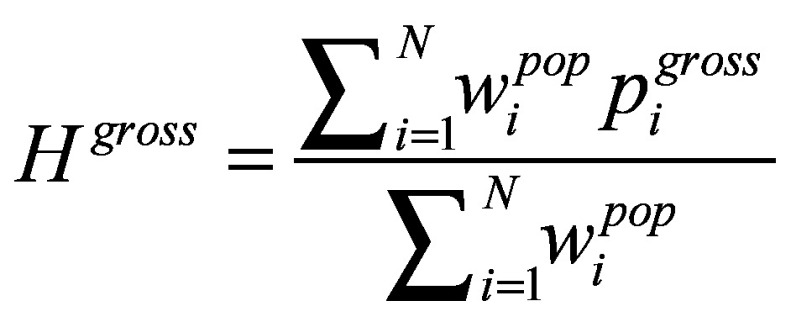
 and 
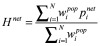
(2)

The share of the population being pushed under the poverty line due to out-of-pocket expenditure, therefore, can be captured as *H^oop^* = *H^net^*−*H^gross^.*

Similarly, 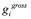
defined as 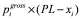, captures the distance of household *i* in its per capita expenditure away from the poverty line, conditional on being under the poverty line, and 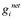, defined as 
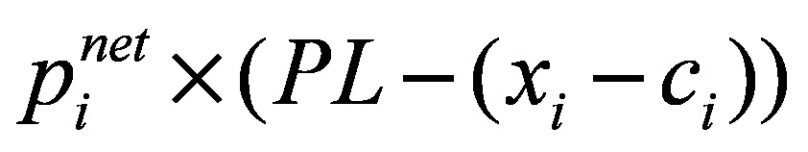
, is similar to 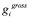 except that per capita expenditure excludes health-care payments. Then *G^gross^*, or gross poverty gap, and *G^net^,* net poverty gap, are defined in Equation 3:


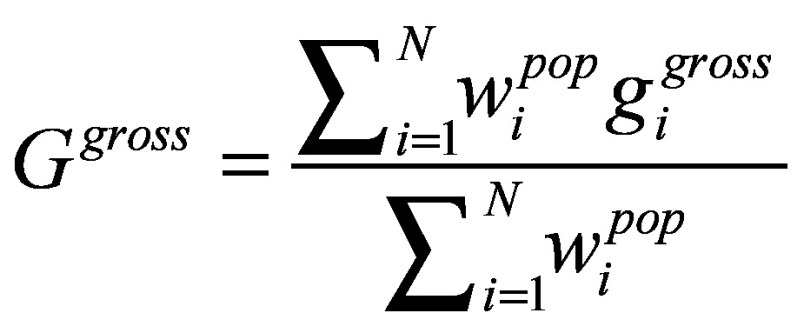
 and 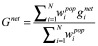
(3)

And the difference between the two, *G^oop^* = *G^net^*−*G^gross^*, measures the change in poverty gaps due to out-of-pocket payment on health, expressed as the percentage of poverty lines in this paper.

To determine the main drivers of out-of-pocket expenditure, we decomposed it by categories of spending and analysed their relative size. Suppose 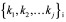 is expenditure by household *i* on component of *k_1_*,* k_2_*,* … k_j_*, and let *k_1_* be out-of-pocket expenditure on medicines as that is universally available of all surveys, while, *k_2_*,* … k_j_* might represent different items across countries. Then the average share of out-of-pocket spending on each component can be defined in Equation 4:


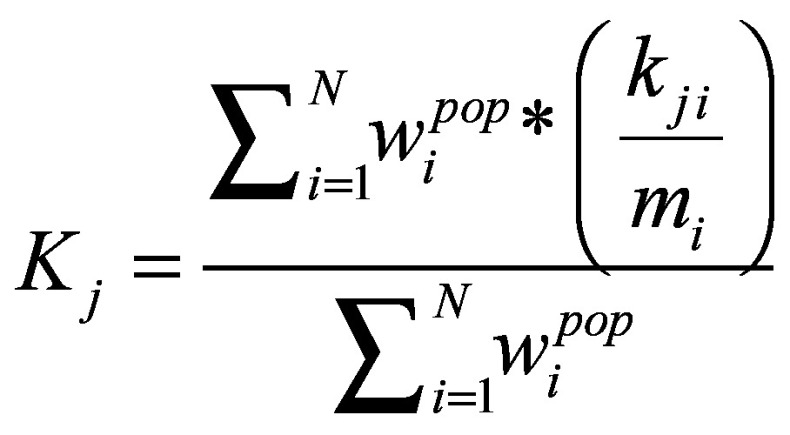
(4)

When *j* = 1, the above equation measures the average share of out-of-pocket spending on medicines.

### Data sources

We included eight countries of the WHO South-East Asia Region in the study: Bangladesh, Bhutan, India, Maldives, Nepal, Sri Lanka, Thailand and Timor-Leste. We did not include Indonesia as their survey instrument was recognized as being unable to separate actual out-of-pocket spending from insurance reimbursement. We also excluded Democratic Republic of Korea and Myanmar, because to our knowledge there were no national surveys at the time that met the criteria for our analysis. We used data from the most recently available household surveys in each country, which were either living standards and measurement surveys or household income and expenditure surveys (Table 1). These are the most appropriate types of survey for such analysis, because they are nationally representative and have a detailed documentation of household consumption, including that of health care. Some of these surveys have also been used to estimate national poverty ratios and many have been used for National Health Accounts for the estimate of out-of-pocket expenditure.^18^ The full lists of variables in each data set used for the analysis are listed in Table 2 (available at: http://www.who.int/bulletin/volumes/96/9/18-209858).

**Table 1:** Type and year of survey in countries included in the financial protection analysis in the South-East Asia Region

Country	Survey year	Total population in the survey year^a^	Survey type	Sample size, no. of households	No. of households responding (%)
Bangladesh	2010	152 149 102	Household income and expenditure survey	12 239	12 239 (100)
Bhutan	2012	752 967	Living standards survey	8 968	8 699 (97)
India	2011	1 247 236 029	Household consumer expenditure survey	101 662	101 662 (100)
Maldives	2009	354 501	Household income and expenditure survey	1 917	1 783 (93)
Nepal	2014	28 323 241	Annual household survey	4 320	4 147 (96)
Sri Lanka	2012	20 425 000	Household income and spending survey	20 540	16 637 (81)
Thailand	2015	68 657 600	Household socioeconomic survey	43 400	36 022 (83)
Timor-Leste	2014	1 212 814	Household expenditure survey	5 916	5 916 (100)

^a^ Data source: World Development Indicators.^17^

Notes: All studies had a stratified study design with weightings applied to make the results nationally representative.

**Table 2:** List of variables in survey data sets in the financial protection analysis of eight countries in the South-East Asia Region

Country	Demographic information: used in all analysis	Household expenditure on health	Components of health expenditure	Household expenditure on everything except health
Bangladesh	• Household weight: wgt • Rural/urban classification: spc • Household size: idcode	• s03a__17	• s03a_q_9	Food: o s09a1d_2, s09a1d_5, s09a1d_8, s09a1_11, s09a1_14, s09a1_17, s09a1_20, s09a1_23, s09a1_26, s09a1_29, s09a1_32, s09a1_35, s09a1_38, s09a1_41 o s09b1w_2, s09b1w_5 Nonfood:o s09c1__2, s09d1__1, s09d2_q0 o s02b__15
Bhutan	• Household weight: weight • Rural/urban classification: area • Household size: hsize	• h5a, h5b, h5f, h5 g • h10a, h10b, h10f, h10 g • h7 • f10,h f10i, f10m, f10n	• Medicine: h5b, h10b, f10i, h7 • Hospital charges/consultation fees: h5a, h10a, f10h • Traditional practices: h5f, h10f, f10m • Others: h5 g, h10 g, f10n	• Food: fc9, feb, fec • Nonfood: o rm12, rm13, rm22, rm23, rm31, rm32, rm41, rm42, rm51, rm52 o hs6 o hs30a – hs30f, hs32b, hs33b, hs35a – hs35 g, hs36 o ed8a1 – ed8a6, ed8b1 – ed8b6 o nf2, nf4
India	• Household weight: wgt • Rural/urban classification: sector • Household size: hsize	• oop1 (iptotal1, optotal)	• Medicine: ip11, op1 • Diagnostic test: ip21, op2 • Doctor’s fees: ip31, op3 • Hospital & nursing home charges and family planning devices: ip41, op4 • Others: ip52, op5	• mpce2 • mce2 • food
Maldives	• Household weight: raising factor • Rural/urban classification: region Household size: poptot	Monthly cost • coicop2	• Medicine: monthly cost when coicop = = ”06.1.1,” “06.1.2,” or “06.1.3” • Outpatient: monthly cost when coicop = = ”06.2.1,” “06.2.2,” or “06.2.3” • Hospital: monthly cost when coicop = = ”06.3.0” • Overseas: monthly cost when coicop = = ”06”	• monthly cost • q131 – q134 • q91amount – q94amount
Nepal	• Household weight: wt_hh_adj • Rural/urban classification: urbrur • Household size: idcode1	• amount12 non-food code	• Medicine: amount12 when non-food code is one of the following: 611, 612, 613 • Outpatient: amount12 when non-food code is one of the following: 621, 622, 623 • Inpatient: amount12 when non-food code is one of the following: 631, 632, 633 • International medicine: amount12 when non-food code is one of the following:1285, 1286 • International service: amount12 when non-food code is 1287	• Food: home_val, purchase_val, received_val, yes/no • Non-food: • amount12, yes/no, non-food code • rent_paid • amt_water, amt_jarwater, amt_tankarwater, amt_waste, amt_light, amt_wood, firewood_price_unit
Sri Lanka	• Household weight: weight • Rural/urban classification: sector • Household size: person_serial_no, member_resident	• nf_value • nf_code	• Medicine: nf_value if nf_code is 2306 • Medical tests: nf_value if nf_code is one of the following: 2304, 2309, 2310 • Consultation fees: nf_value if nf_code is one of the following: 2301, 2302, 230 • Medical equipment: nf_value if nf_code is one of the following: 2307, 2308 • Private hospitals and nursing homes: nf_value if nf_code is 2305 • Others: nf_value if nf_code is 2319	• Food: value, inkind_value • Non-food (excluding health): nf_code, nf_value, col_7 • Servants/boarders: col_4 - col_15
Thailand	• Household weight: A52 • Rural/urban classification: area • Household size: A04	• EG58a, EG58bc, EG59a, EG59bc, EG60a, EG60bc • EG52a, EG52bc, EG53a, EG53bc, EG54a EG54bc, EG55a EG55bc, EG47a EG47bc EG48a EG48bc EG49a EG49bc EG50a EG50bc EG51a EG51bc • EG56bc, EG57a EG57bc	• Medicine: EG58a, EG58bc, EG59a, EG59bc, EG60a, EG60bc • Inpatient: EG52a, EG52bc, EG53a, EG53bc, EG54a EG54bc, EG55a EG55bc, EG47a EG47bc EG48a EG48bc EG49a EG49bc EG50a EG50bc EG51a EG51bc • Outpatient: EG56bc, EG57a EG57bc	• Food: A09, A12 • Non-food: A07
Timor-Leste	• Household weight: hhweight • Rural/urban classification: urban • Household size: q06_06	• q06_21_a – q06_21_d • q06_28 • q06_33 • q06_34 • q06_30	• Medicine: q06_21_b, q06_28 • Inpatient care: q06_33, q06_34, q06_30 • Outpatient care: q06_21_a, q06_21_c, q06_21_d	• Food: q04_03, food_code • Non-food (excluding health): o id_nf o q04_06 o q04_09 o q04_12, q04_13, q04_11_yes, q04_11_no o q05_21_a – q05_21_h, q05_22 o q09_35, q09_36, q09_38, q09_39_1 – q09_39_2 o q10_05 o q02_30, q02_32 o q02_34, q02_35 o q13_04_u, q13_04_us

### Data analysis

We used recall periods of 30 days for outpatient care to reduce bias of recall and 12 months for inpatient care to reduce bias due to infrequent occurrence. To generate total household expenditure on health, we separated out items which, although asked about under health modules, do not belong to health services. These include *rimdo* or *puja* (or religious treatment, in Bhutan) and transport costs. In rare cases when health-care expenditure was asked both in the health and non-food modules of the survey, only the former was counted in out-of-pocket expenditure.

We used the two international recommended thresholds to define large out-of-pocket health expenditure: above 10% and above 25% of total household expenditure or income.^14^^,^^15^ The definition of poverty usually varies across countries, so for comparison we used the two international poverty lines at the time of the study of 1.9 United States dollars (US$) and US$ 3.1 per capita per day (based on 2011 purchasing power parity exchange rates)^19^ to define the incidence of poverty due to out-of-pocket expenditure and the poverty gap.

We grouped national population into five economic quintiles based on their per capita consumption level. We used Stata 2014 (StataCorp LLC, College Station, United States of America) for all analyses.

## Results

**Fig. 1:**
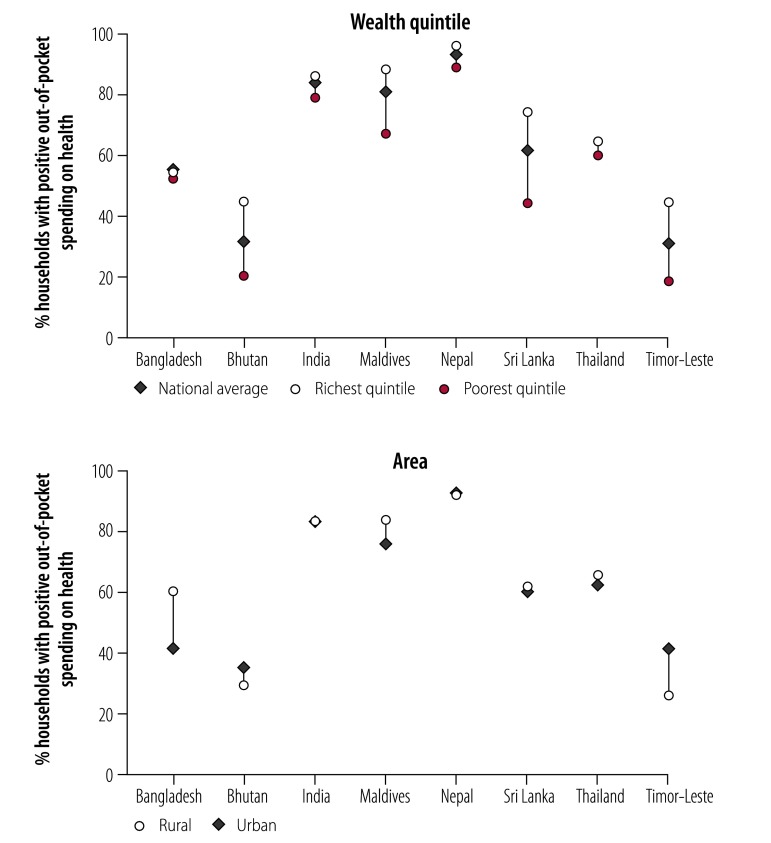
Share of households with positive out-of-pocket spending on health in countries included in the financial protection analysis in the South-East Asia Region, by richest and poorest quintiles and by area

Note: Bars show the difference between the richest and the poorest quintiles.

**Fig. 2:**
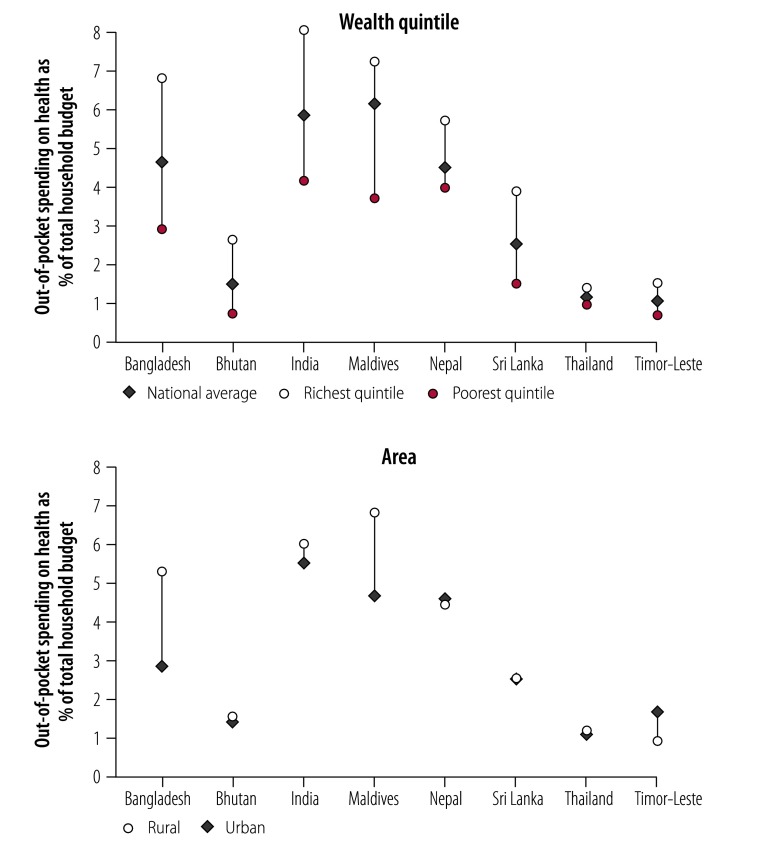
Share of out-of-pocket spending on health as total household budget in countries included in the financial protection analysis in the South-East Asia Region, by richest and poorest quintiles and by area

Note: Bars show the difference between the richest and the poorest quintiles.

Table 3 shows the main sociodemographic and health-system characteristics of the countries analysed. There were large variations in economic development and population health across countries, but a common pattern of heavy reliance of out-of-pocket expenditure. Fig. 1 and Fig. 2 summarize the basic characteristics of out-of-pocket expenditure in each country. On average, in most countries, more than 50% of the population had some level of out-of-pocket expenditure spending. The average ranged from 1.1% to 6.1% of total household budget, or purchasing power parity US$ 1.1–21.9 per capita per month. As expected, richer populations had more out-of-pocket spending and the spending was higher both in absolute (dollars) and relative (% of household budget) measures. There was no consistent pattern between rural and urban households across countries.

**Table 3:** Sociodemographic and health systems characteristics of countries included in the financial protection analysis in the South-East Asia Region

Country	Population thousands in 2016	GDP per capita in 2016, current US$	Urban population in 2016, %	Income group^a^	Life expectancy at birth in 2016, years	Under-five mortality rate in 2016, per 1000 live births	Current health expenditure per capita in 2015, current US$	Domestic general government health expenditure in 2015, % GGE	Out-of-pocket expenditure in 2015, % CHE
Bangladesh	162 952	1 359	35	Lower middle	72	34	32	2.8	74.3
Bhutan	798	2 774	39	Lower middle	70	32	91	9.1	22.6
India	1 324 171	1 710	33	Lower middle	69	43	63	3.4	73.5
Maldives	428	9 875	47	Upper middle	77	9	944	22.8	18.0
Nepal	28 983	729	19	Low	70	35	44	5.5	71.4
Sri Lanka	21 203	3 835	18	Lower middle	75	9	118	7.9	45.2
Thailand	68 864	5 911	52	Upper middle	75	12	219	15.3	23.9
Timor-Leste	1 269	1 405	33	Lower middle	69	50	72	4.2	10.3

CHE: current health expenditure; GDP: gross domestic product; GGE: general government expenditure; US$: United States dollars.

^a^ World Bank classification.^20^

Data source: World Development Indicators.^17^ Global Health Expenditure Database.^4^

### Catastrophic health spending

Table 4 (available at: http://www.who.int/bulletin/volumes/96/9/18-209858) presents the incidence of catastrophic health expenditure. For both thresholds, Maldives had the highest share of the population experiencing catastrophic health expenditures, followed by India and Bangladesh. Thailand and Timor-Leste had the lowest (at the 10% poverty threshold). Based on the total populations reported in the corresponding survey years (Table 1), we estimated that across the eight countries, 242.7 million people had catastrophic expenditure at the 10% threshold and 56.4 million at the 25% threshold.

**Table 4:** Incidence of catastrophic spending on health at two different thresholds of total household expenditure in countries included in the financial protection analysis in the South-East Asia Region

Country, by variable	National average	Quintile					Area	
		Poorest	Poorer	Middle	Richer	Richest	Rural	Urban
**10% threshold^a^**								
Bangladesh								
Incidence of catastrophic spending, % (SE)	13.86 (0.36)	8.54 (0.68)	11.59 (0.77)	13.44 (0.79)	17.78 (0.91)	17.95 (0.89)	15.85 (0.45)	8.27 (0.52)
No. of people with catastrophic spending, thousands	21 088	2 599	3 527	4 090	5 410	5 462	17 777	3 307
Bhutan								
Incidence of catastrophic spending, % (SE)	4.06 (0.26)	1.89 (0.52)	3.23 (0.63)	3.82 (0.57)	4.21 (0.55)	6.86 (0.65)	4.30 (0.36)	3.54 (0.31)
No. of people with catastrophic spending, thousands	31	3	5	6	6	10	22	8
India								
Incidence of catastrophic spending, % (SE)	17.32 (0.25)	11.20 (0.58)	13.33 (0.55)	16.89 (0.59)	21.14 (0.57)	24.04 (0.49)	17.81 (0.32)	16.10 (0.34)
No. of people with catastrophic spending, thousands	216 021	27 938	33 251	42 132	52 733	59 967	158 665	57 374
Maldives								
Incidence of catastrophic spending, % (SE)	19.88 (1.40)	12.99 (3.50)	14.19 (2.55)	25.97 (3.68)	23.42 (3.21)	22.83 (2.43)	22.81 (1.90)	13.83 (1.66)
No. of people with catastrophic spending, thousands	70	9	10	18	17	16	54	16
Nepal								
Incidence of catastrophic spending, % (SE)	10.71 (0.56)	8.49 (1.38)	8.66 (1.19)	10.12 (1.21)	11.77 (1.21)	14.54 (1.22)	10.19 (0.71)	11.93 (0.84)
No. of people with catastrophic spending, thousands	3 033	481	491	573	667	824	2 017	1 018
Sri Lanka								
Incidence of catastrophic spending, % (SE)	5.33 (0.18)	2.84 (0.34)	3.71 (0.37)	4.84 (0.40)	6.49 (0.43)	8.79 (0.48)	5.35 (0.21)	5.27 (0.37)
No. of people with catastrophic spending, thousands	1 089	116	152	198	265	359	897	193
Thailand								
Incidence of catastrophic spending, % (SE)	1.88 (0.10)	1.63 (0.21)	1.51 (0.17)	1.58 (0.18)	1.78 (0.21)	2.70 (0.26)	1.87 (0.13)	1.88 (0.14)
No. of people with catastrophic spending, thousands	1 291	224	207	217	244	371	717	570
Timor-Leste								
Incidence of catastrophic spending, % (SE)	2.93 (0.44)	1.78 (0.68)	1.34 (0.48)	3.77 (1.59)	3.94 (1.02)	3.81 (0.81)	1.93 (0.27)	5.46 (1.38)
No. of people with catastrophic spending, thousands	35.54	4.32	3.25	9.14	9.56	9.24	16.80	18.70
**25% threshold^a^**								
Bangladesh								
Incidence of catastrophic spending, % (SE)	4.39 (0.22)	0.93 (0.23)	1.90 (0.32)	3.36 (0.42)	6.18 (0.59)	9.58 (0.68)	5.10 (0.27)	2.39 (0.30)
No. of people with catastrophic spending, thousands	6 679	283	578	1 022	1 881	2 915	5 720	956
Bhutan								
Incidence of catastrophic spending, % (SE)	1.45 (0.16)	0.56 (0.30)	0.78 (0.33)	1.33 (0.35)	1.22 (0.30)	3.04 (0.45)	1.59 (0.22)	1.14 (0.19)
No. of people with catastrophic spending, thousands	11	1	1	2	2	5	8	3
India								
Incidence of catastrophic spending, % (SE)	3.89 (0.12)	0.81 (0.16)	2.06 (0.24)	3.19 (0.26)	5.13 (0.32)	8.28 (0.34)	4.16 (0.16)	3.22 (0.16)
No. of people with catastrophic spending, thousands	48 517	2 021	5 139	7 957	12 797	20 654	37 060	11 475
Maldives								
Incidence of catastrophic spending, % (SE)	6.17 (0.86)	1.65 (0.89)	1.75 (1.01)	9.76 (2.90)	8.23 (2.11)	9.46 (1.72)	7.37 (1.19)	3.68 (0.88)
No. of people with catastrophic spending, thousands	22	1	1	7	6	7	18	4
Nepal								
Incidence of catastrophic spending, % (SE)	2.41 (0.27)	0.92 (0.48)	0.95 (0.40)	2.35 (0.62)	2.62 (0.61)	5.23 (0.78)	2.39 (0.35)	2.47 (0.36)
No. of people with catastrophic spending, thousands	683	52	54	133	148	296	473	211
Sri Lanka								
Incidence of catastrophic spending, % (SE)	0.91 (0.08)	0.18 (0.08)	0.31 (0.09)	0.35 (0.11)	1.02 (0.18)	2.72 (0.28)	0.93 (0.09)	0.86 (0.16)
No. of people with catastrophic spending, thousands	186	7	13	14	42	111	156	31
Thailand								
Incidence of catastrophic spending, % (SE)	0.36 (0.04)	0.23 (0.08)	0.39 (0.10)	0.26 (0.07)	0.32 (0.07)	0.55 (0.12)	0.36 (0.05)	0.36 (0.07)
No. of people with catastrophic spending, thousands	247	32	54	36	44	76	138	109
Timor-Leste								
Incidence of catastrophic spending, % (SE)	0.50 (0.11)	0.47 (0.29)	0.36 (0.22)	0.14 (0.10)	0.75 (0.35)	0.78 (0.24)	0.53 (0.15)	0.43 (0.15)
No. of people with catastrophic spending, thousands	6	1	1	0	2	2	5	1

SE: standard error.

^a^ Thresholds are the proportion of the population spending more than 10% or 25% of their total household expenditure on health.^14^^,^^15^

The finding that poorer households had lower incidence of catastrophic health spending is consistent with global studies, and is aligned with the above findings that poorer households spent less on health care, both in absolute and relative terms (Fig. 1 and Fig. 2). The pattern across rural versus urban areas was less clear, with the incidence of catastrophic spending much higher in rural than urban areas in Bangladesh, Bhutan, India and Maldives.

When capacity-to-pay was used as an alternative denominator, we found very few people made more than 40% of their non-subsistence spending on health, and the richest quintile was still more likely to spend a bigger share of their budget on health (data are available from the corresponding author).

### Impoverishing health spending

Table 5 shows the impoverishing effect of health-care spending expressed as the share of the population being pushed below the poverty line. In total 58.2 million people were pushed below the extreme poverty line of purchasing power parity US$ 1.90 per capita per day and 64.2 million below the poverty line of US$ 3.10. India and Bangladesh had the highest share of the population affected, translating into 52.5 million and 5.2 million people, respectively, being pushed under the US$ 1.90 poverty line. When US$ 3.10 was used as the poverty line, another two countries, Maldives and Nepal, were also affected. In both cases, Thailand had the fewest people impoverished due to out-of-pocket spending.

**Table 5:** Share of the population being pushed below two different poverty lines due to out-of-pocket expenditure in countries included the financial protection analysis in the South-East Asia Region

Country, by variable	National average	Quintile					Area	
		Poorest	Poorer	Middle	Richer	Richest	Rural	Urban
**Poverty line US$ 1.90^a^**								
Bangladesh								
% of population under poverty line (SE)	3.44 (0.20)	0.00 (NA)	13.93 (0.83)	1.91 (0.32)	0.73 (0.24)	0.61 (0.20)	4.15 (0.25)	1.44 (0.23)
No. of people pushed below poverty line	5 234	0	4 239	581	222	186	4 655	576
Bhutan								
% of population under poverty line (SE)	0.32 (0.09)	1.31 (0.42)	0.28 (0.20)	0.00 (NA)	0.00 (NA)	0.00 (NA)	0.43 (0.13)	0.06 (0.04)
No. of people pushed below poverty line	2	2	0	0	0	0	2	0
India								
% of population under poverty line (SE)	4.21 (0.16)	0.00 (NA)	17.61 (0.66)	2.49 (0.24)	0.67 (0.13)	0.25 (0.11)	5.24 (0.21)	1.61 (0.11)
No. of people pushed below poverty line	52 509	0	43 928	6 211	1 671	624	46 682	5 737
Maldives								
% of population under poverty line (SE)	1.49 (0.51)	7.34 (2.42)	0.00 (NA)	0.00 (NA)	0.00 (NA)	0.00 (NA)	2.12 (0.75)	0.17 (0.17)
No. of people pushed below poverty line	5	5	0	0	0	0	5	0
Nepal								
% of population under poverty line (SE)	1.67 (0.25)	6.90 (1.13)	0.97 (0.39)	0.46 (0.27)	0.00 (NA)	0.00 (NA)	1.98 (0.34)	0.94 (0.25)
No. of people pushed below poverty line	473	391	55	26	0	0	392	80
Sri Lanka								
% of population under poverty line (SE)	0.07 (0.02)	0.34 (0.11)	0.00 (NA)	0.00 (NA)	0.00 (NA)	0.00 (NA)	0.08 (0.03)	0.00 (NA)
No. of people pushed below poverty line	14	14	0	0	0	0	14	0
Thailand								
% of population under poverty line (SE)	0.00 (NA)	0.00 (NA)	0.00 (NA)	0.00 (NA)	0.00 (NA)	0.00 (NA)	0.00 (NA)	0.00 (NA)
No. of people pushed below poverty line	0	0	0	0	0	0	0	0
Timor-Leste								
% of population under poverty line (SE)	0.99 (0.33)	0.00 (NA)	0.00 (NA)	4.67 (1.61)	0.17 (0.13)	0.10 (0.07)	0.79 (0.19)	1.50 (1.08)
No. of people pushed below poverty line	12	0	0	11	0	0	7	5
**Poverty line US$ 3.10^a^**								
Bangladesh								
% of population under poverty line (SE)	4.06 (0.21)	0.00 (NA)	0.00 (NA)	5.94 (0.56)	11.75 (0.76)	2.63 (0.37)	4.57 (0.26)	2.65 (0.32)
No. of people pushed below poverty line	6 177	0	0	1 808	3 576	800	5 126	1 060
Bhutan								
% of population under poverty line (SE)	0.93 (0.15)	0.00 (NA)	3.28 (0.65)	1.16 (0.32)	0.13 (0.09)	0.07 (0.07)	1.18 (0.21)	0.36 (0.10)
No. of people pushed below poverty line	7	0	5	2	0	0	6	1
India								
% of population under poverty line (SE)	4.56 (0.14)	0.00 (NA)	0.00 (NA)	0.00 (NA)	21.11 (0.57)	1.71 (0.20)	4.86 (0.17)	3.83 (0.20)
No. of people pushed below poverty line	56 874	0	0	0	52 658	4 266	43 297	13 649
Maldives								
% of population under poverty line (SE)	3.03 (0.68)	0.00 (NA)	9.75 (2.28)	4.39 (2.39)	1.14 (0.59)	0.00 (NA)	4.15 (0.99)	0.73 (0.43)
No. of people pushed below poverty line	11	0	7	3	1	0	10	1
Nepal								
% of population under poverty line (SE)	3.44 (0.33)	0.00 (NA)	0.00 (NA)	15.50 (1.48)	1.51 (0.43)	0.21 (0.16)	3.79 (0.44)	2.64 (0.39)
No. of people pushed below poverty line	974	0	0	878	86	12	750	225
Sri Lanka								
% of population under poverty line (SE)	0.83 (0.08)	3.86 (0.40)	0.26 (0.10)	0.00 (NA)	0.00 (NA)	0.04 (0.04)	0.97 (0.10)	0.19 (0.08)
No. of people pushed below poverty line	170	158	11	0	0	2	163	7
Thailand								
% of population under poverty line (SE)	< 0.01 (< 0.00)	0.02 (0.02)	0.00 (NA)	0.00 (NA)	0.00 (NA)	0.00 (NA)	< 0.01 (< 0.00)	< 0.01 (< 0.00)
No. of people pushed below poverty line	2	2	0	0	0	0	1.0	1.1
Timor-Leste								
% of population under poverty line (SE)	0.64 (0.13)	0.00 (NA)	0.00 (NA)	0.00 (NA)	2.85 (0.61)	0.36 (0.16)	0.45 (0.13)	1.13 (0.31)
No. of people pushed below poverty line	8	0	0	0	7	1	4	4

NA: not applicable; SE: standard error: US$: United States dollars.

^a^ Poverty lines are expressed as purchasing power parity per capita per day with exchange rates based on World Development Indicators.^17^

Notes: The number 0 in the poorer quintiles mean that all people were already below poverty lines and could be pushed below them again due to health expenditure. In other words, they are vulnerable regardless of outcome. In contrast, the 0 observed in richer quintiles mean that no one in the group was pushed below poverty lines due to health expenditure and are therefore not vulnerable.

It is worth noting that the value of zero in Table 5, mostly observed in the lowest quintiles, represented those who were already classified as poor; as a result, any out-of-pocket expenditure on health care would only further their financial hardship. Given this, the data clearly show that the poorer suffer much more than their richer counterparts. A typical example is Timor-Leste, where the poorest 40% of the population (at the US$ 1.90 poverty line) were vulnerable to further impoverishment by out-of-pocket expenditure on health. Coupled with the very low out-of-pocket expenditure in Timor-Leste (Fig. 1 and Fig. 2), the results show that the poor have limited capacity to cope with any out-of-pocket expenditure on health.

Analysis of the changes in poverty gaps induced by out-of-pocket expenditure showed that the impact was highest in Nepal (Table 6; available at: http://www.who.int/bulletin/volumes/96/9/18-209858) indicating that the out-of-pocket expenditure pushed people not only below, but also further away from the poverty lines.

**Table 6:** Changes in the poverty gaps due to out-of-pocket health expenditures at two different poverty lines in countries included in the financial protection analysis in the South-East Asia Region

Country	Change in poverty gaps due to out-of-pocket health expenditures, % (SE)							
	National average	Quintiles					Area	
		Poorest	Poorer	Middle	Richer	Richest	Rural	Urban
**Poverty line US$ 1.90^a^**								
Bangladesh	0.81 (0.04)	2.40 (0.11)	1.22 (0.11)	0.27 (0.06)	0.07 (0.03)	0.12 (0.05)	1.02 (0.05)	0.24 (0.03)
Bhutan	0.10 (0.02)	0.43 (0.10)	0.06 (0.04)	0.00 (NA)	0.00 (NA)	0.00 (NA)	0.14 (0.03)	0.01 (0.00)
India	1.16 (0.03)	3.22 (0.08)	2.18 (0.09)	0.26 (0.03)	0.09 (0.02)	0.07 (0.04)	1.47 (0.04)	0.39 (0.02)
Maldives	0.23 (0.06)	1.13 (0.29)	0.00 (NA)	0.00 (NA)	0.00 (NA)	0.00 (NA)	0.32 (0.09)	0.04 (0.03)
Nepal	3.97 (0.24)	19.73 (0.85)	0.09 (0.05)	0.01 (0.01)	0.00 (NA)	0.00 (NA)	4.78 (0.32)	2.09 (0.3)
Sri Lanka	0.02 (< 0.01)	0.07 (0.02)	0.00 (NA)	0.00 (NA)	0.00 (NA)	0.00 (NA)	0.02 (< 0.01)	< 0.01 (< 0.01)
Thailand	< 0.01 (< 0.01)	< 0.01 (< 0.01)	0.00 (NA)	0.00 (NA)	0.00 (NA)	0.00 (NA)	< 0.01 (< 0.01)	0.00 (NA)
Timor-Leste	0.32 (0.07)	0.37 (0.08)	0.63 (0.09)	0.59 (0.33)	0.02 (0.01)	0.01 (0.00)	0.26 (0.03)	0.47(0.24)
**Poverty line US$ 3.10^a^**								
Bangladesh	2.05 (0.06)	1.47 (0.07)	2.57 (0.11)	3.78 (0.17)	1.79 (0.17)	0.65 (0.12)	2.49 (0.08)	0.82 (0.05)
Bhutan	0.29 (0.03)	0.46 (0.08)	0.88 (0.12)	0.10 (0.04)	< 0.01 (< 0.01)	< 0.01 (< 0.01)	0.39 (0.04)	0.08 (0.01)
India	2.51 (0.04)	1.97 (0.05)	3.21 (0.07)	4.85 (0.09)	2.16 (0.10)	0.35 (0.07)	2.97 (0.05)	1.36 (0.03)
Maldives	0.77 (0.12)	2.70 (0.52)	0.77 (0.16)	0.19 (0.11)	0.15 (0.09)	0.00 (NA)	1.03 (0.18)	0.23 (0.06)
Nepal	1.43 (0.07)	2.06 (0.13)	3.19 (0.18)	1.69 (0.21)	0.17 (0.06)	0.06 (0.04)	1.67 (0.09)	0.89 (0.07)
Sri Lanka	0.15 (0.01)	0.71 (0.04)	0.02 (< 0.01)	0.00 (NA)	0.00 (NA)	0.01 (0.01)	0.17 (0.01)	0.05 (0.01)
Thailand	< 0.01 (< 0.01)	< 0.01 (< 0.01)	< 0.01 (< 0.01)	< 0.01 (< 0.01)	0.00 (NA)	0.00 (NA)	< 0.01 (< 0.01)	< 0.01 (< 0.01)
Timor-Leste	0.54 (0.06)	0.22 (0.05)	0.38 (0.06)	0.84 (0.21)	1.13 (0.17)	0.11 (0.05)	0.45 (0.04)	0.75 (0.18)

NA: not applicable; SE: standard error: US$: United States dollars.

^a^ Poverty lines (thresholds) are US$ purchasing power parity per capita per day with exchange rates based on World Development Indicators.^17^

### Drivers of out-of-pocket spending

**Fig. 3:**
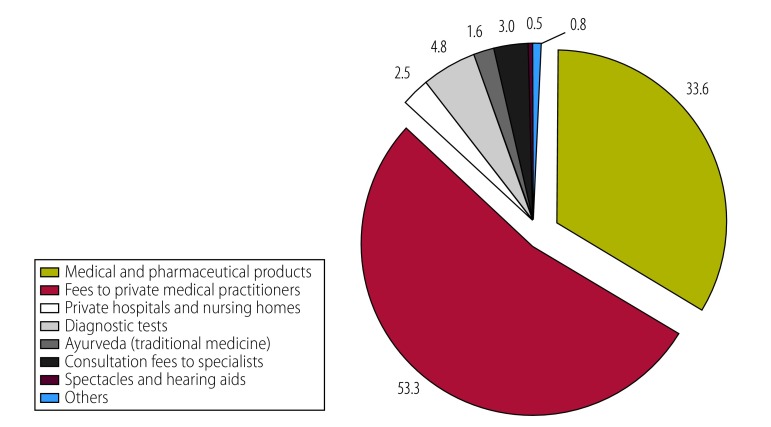
Components of out-of-pocket spending on health in Sri Lanka

Notes: The chart shows percentage of total out of-pocket spending on different components of health. Data are from 2009.

**Fig. 4:**
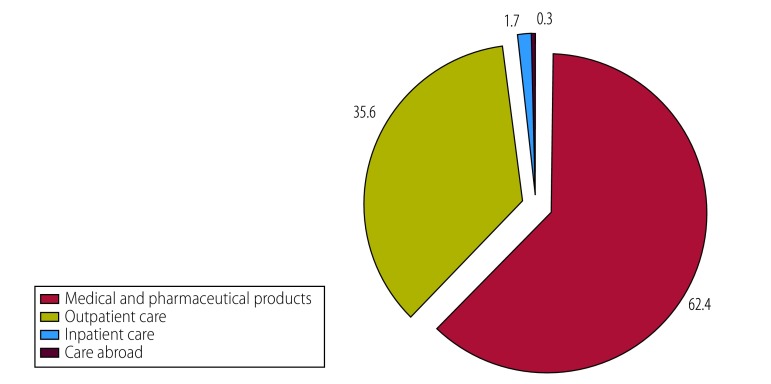
Components of out-of-pocket spending on health in Maldives

Notes: The chart shows percentage of total out of-pocket spending on different components of health. Data are from 2012.

Spending on medicines was the dominant component of out-of-pocket expenditure on health care in all countries except Sri Lanka (Table 7). Moreover, in all except two countries the share of out-of-pocket expenditure due to medicines exceeded 70%. In the two exceptions, other important out-of-pocket expenditure components included fees paid to private medical practitioners in Sri Lanka (Fig. 3) and outpatient visits in Maldives (Fig. 4). In general, poorer households spent relatively more on medicines than did their richer counterparts. Data on all health expenditures are available from the corresponding author.

**Table 7:** Share of out-of-pocket health expenditure on medicines in countries included in the financial protection analysis in the South-East Asia Region

Country	Share of out-of-pocket health expenditure, % (SE)							
	National average	Quintiles					Region	
		Poorest	Poorer	Middle	Richer	Richest	Rural	Urban
Bangladesh	81.09 (0.37)	89.41 (0.65)	85.41 (0.70)	82.12 (0.83)	77.62 (0.82)	71.09 (0.93)	82.02 (0.39)	77.33 (0.92)
Bhutan	76.38 (0.86)	69.23 (3.44)	72.44 (2.42)	77.02 (2.11)	80.80 (1.68)	78.19 (1.40)	69.31 (1.25)	89.39 (0.76)
India	79.93 (0.17)	85.30 (0.33)	81.24 (0.36)	78.46 (0.34)	74.69 (0.38)	71.98 (0.39)	81.51 (0.21)	75.96 (0.26)
Maldives	62.37 (1.39)	71.25 (3.30)	57.66 (3.33)	64.28 (3.21)	62.74 (2.47)	57.73 (2.88)	65.80 (1.72)	54.52 (2.17)
Nepal	77.13 (0.53)	85.15 (1.26)	79.31 (1.23)	78.06 (1.13)	72.41 (1.19)	71.36 (0.95)	77.08 (0.69)	77.25 (0.69)
Sri Lanka	34.05 (0.44)	35.66 (1.35)	31.64 (1.07)	35.07 (1.00)	34.10 (0.88)	34.04 (0.77)	32.53 (0.49)	41.30 (0.94)
Thailand	75.06 (0.37)	82.10 (0.91)	78.31 (0.76)	74.35 (0.81)	73.75 (0.79)	70.37 (0.87)	73.43 (0.53)	77.22 (0.51)
Timor-Leste	81.89 (1.11)	80.19 (3.56)	78.58 (2.98)	86.37 (2.21)	82.22 (2.15)	81.08 (2.07)	81.74(1.44)	82.13 (1.72)

SE: standard error.

## Discussion

Using the latest available surveys, our study provides a cross-sectional description of financial protection against out-of-pocket expenditure for eight countries in WHO South-East Asia Region, with two key findings. First, most countries (except Thailand, Sri Lanka and Timor-Leste) performed below the global median rate for at least one of the indicators.^15^ Second, the dominant role of out-of-pocket expenditure on medicines has been observed over the past decade in the Region, as corroborated by earlier studies.^21^^–^^25^ For instance, medicines constituted 72% of total out-of-pocket expenditure payments in India as early as 2004,^21^ almost unchanged until 2011.

Our findings suggest that more effective health policies are needed to provide better financial protection of households. Several attempts have already been made in the Region. In India, the *Rashtriya Swasthya Bima Yojana* scheme was launched in 2008 to provide protection for households below the poverty line. Despite a more than twofold increase in enrolment on average from 2011 to 2016, there were still large gaps in coverage in some states and generosity of benefit packages varied across states.^26^^,^^27^ Furthermore, evidence suggested that even for the insured households, *Rashtriya Swasthya Bima Yojana* did not affect either the likelihood or the level of out-of-pocket expenditure spending, thus rendering no financial protection for the most vulnerable populations.^27^ By contrast, in Thailand, there was quick expansion of the universal coverage scheme in 2001 to cover the informal sector, with comprehensive inpatient and outpatient health care included in the benefit package. The initiative is one of the reasons behind the successful financial protection of the entire population of Thailand that has been consistently observed.^28^^–^^30^ Maldives had one of the highest levels of catastrophic spending and impoverishment in our study. However, the data represented the situation in year 2009, preceding the launch of the national health insurance programme, *Aasandha*, in 2012. It would be worth reassessing the financial burden of households against out-of-pocket expenditure in Maldives with data from the 2016 household survey, to see if the nationwide insurance scheme has made a difference.

The high financial burden of medicine expenditure found in our study, draws attention to the limitations of current pharmaceutical policies in reducing out-of-pocket expenditure in these countries. All eight countries have defined and regularly updated their essential medicines list and state their intention to provide medicines free-of-charge in public health-care facilities. However, other studies found that, for several reasons, most people in South-East Asian countries purchased medicines from private pharmacies,^31^ exposing themselves to higher risk of financial burden. Studies reported that the poor were more likely to be deterred by the perceived high prices.^32^^,^^33^ This is particularly worrisome as the burden of noncommunicable diseases, which are associated with higher out-of-pocket expenditure and catastrophic health expenditure^22^^,^^34^, is increasing fast in the Region.

While the analysis of impoverishment clearly demonstrated the higher financial burden on poorer households, the incidence of catastrophic expenditure was higher among richer people. This finding is consistent with those from earlier studies in the South-East Asia Region.^14^^,^^15^^,^^35^^–^^38^ The distribution is sensitive to the ways the denominator and household quintiles are constructed. Using the capacity-to-pay approach by deducting food expenditure from household budget is more likely to result in a higher incidence among the poor.^38^ A recent study in Bangladesh found a pro-poor distribution of catastrophic spending by using household assets instead of consumption to determine quintiles. However, the results varied by area (rural versus urban) and the threshold selected for the analysis.^39^

The lack of robustness of the indicator of catastrophic health spending shows its limitation in conveying policy relevant messages (i.e. the poor suffer more and need more targeted policies). This is partly due to the fact that catastrophic health spending is conditional on being able to spend on health care in the first place, omitting people who cannot afford the medical service at all, which is more likely for very poor households.

Both the financial protection indicators we used have other limitations. First, they do not capture indirect costs associated with illness, such as income loss due to disability. Second, they do not differentiate the households who borrow or reduce their savings to compensate for health care. These households may not have been identified as facing financial hardship due to out-of-pocket expenditure in the short-term, but will be economically worse off in the medium term. Therefore, more research is needed to refine the approach.

Variations in the designs of their respective household surveys present a challenge in directly comparing financial protection status across countries. Questions about health spending did not follow the same structure, with different levels of detail and different groupings of out-of-pocket spending components. The former may overestimate health expenditure^40^ while the latter creates difficulty in accurately attributing out-of-pocket expenditure to particular items. The household income and expenditure survey of Sri Lanka is a case in point, where the survey design made it impossible to classify out-of-pocket expenditure into typical inpatient and outpatient care. In addition, the Sri Lanka survey captured out-of-pocket expenditure of both the main households and servants living with the families without further details beyond a lump-sum reporting for the latter. For reporting purposes we assumed that the two groups shared exactly the same structures across out-of-pocket expenditure components, which is unlikely to be true, but is reasonable given its marginal magnitude. Similarly, surveys also varied in the questions asked about non-food, non-health expenditures, both in terms of the type and level of detail of data collected. The poor usually have a larger proportion of spending on food than other categories. Therefore an under- or overestimate of non-food expenditures may impact on the calculation of the denominator, thus affecting how both the financial protection indicators vary across economic quintiles. Finally, while the majority of countries used a mix of recall periods, Nepal’s survey followed a recall period of 12 months. The bias introduced by a long recall period is already well documented.^40^^,^^41^ A standardization of such surveys, such as following the structure of the Classification Of Individual Consumption According to Purpose,^42^ would better support cross-country comparisons.

Despite the limitations, our findings revealed the low-ranking financial protection status of countries in South-East Asia Region and the persistent burden on households from pharmaceutical spending. With the expected increase in demand for health care due to epidemiological and demographic changes, both financing and service delivery policies need to adapt for satisfactory progress towards UHC. We also call for further research efforts to refine the indicators for better monitoring of financial protection and a better reflection of the equity dimension.
