# Corrigendum

**DOI:** 10.2471/BLT.18.101218

**Published:** 2018-12-01

In: Wang H, Vinyals Torres L, Travis P. Financial protection analysis in eight countries in the WHO South-East Asia Region. Bull World Health Organ. 2018 Sep 1;96(9):610–20E. http://dx.doi.org/10.2471/BLT.18.209858

on page 612, Table 3., should be as follows: 

**Table 3:** Sociodemographic and health systems characteristics of countries included in the financial protection analysis in the South-East Asia Region

Country	Population thousands in 2016	GDP per capita in 2016, current US$	Urban population in 2016, %	Income group^a^	Life expectancy at birth in 2016, years	Under-five mortality rate in 2016, per 1000 live births	Current health expenditure per capita in 2015, current US$	Domestic general government health expenditure in 2015, % GGE	Out-of-pocket expenditure in 2015, % CHE
Bangladesh	162 952	1 359	35	Lower middle	72	34	32	2.8	70
Bhutan	798	2 774	39	Lower middle	70	32	91	9.1	20
India	1 324 171	1 710	33	Lower middle	69	43	63	3.4	65
Maldives	428	9 875	47	Upper middle	77	9	944	22.8	16
Nepal	28 983	729	19	Low	70	35	44	5.5	60
Sri Lanka	21 203	3 835	18	Lower middle	75	9	118	7.9	38
Thailand	68 864	5 911	52	Upper middle	75	12	219	15.3	12
Timor-Leste	1 269	1 405	33	Lower middle	69	50	72	4.2	10

CHE: current health expenditure; GDP: gross domestic product; GGE: general government expenditure; US$: United States dollars.

a World Bank classification.

Data source: World Development Indicators. Global Health Expenditure Database.

